# CRISPR/Cas9 gene editing in a chicken model: current approaches and applications

**DOI:** 10.1007/s13353-020-00537-9

**Published:** 2020-01-10

**Authors:** Luiza Chojnacka-Puchta, Dorota Sawicka

**Affiliations:** 1 Department of Bioengineering, Lukasiewicz Research Network, Institute of Biotechnology and Antibiotics, Staroscinska 5, 02-516 Warsaw, Poland; 2 Department of Chemical, Biological and Aerosol Hazards, Central Institute for Labour Protection-National Research Institute, Czerniakowska 16, 00-701 Warsaw, Poland

**Keywords:** CRISPR/Cas9 system, Primordial germ cells, Gesicle technology, Chicken

## Abstract

Improvements in genome editing technology in birds using primordial germ cells (PGCs) have made the development of innovative era genome-edited avian models possible, including specific chicken bioreactors, production of knock-in/out chickens, low-allergenicity eggs, and disease-resistance models. New strategies, including CRISPR/Cas9, have made gene editing easy and highly efficient in comparison to the well-known process of homologous recombination. The clustered regularly interspaced short palindromic repeats (CRISPR) technique enables us to understand the function of genes and/or to modify the animal phenotype to fit a specific scientific or production target. To facilitate chicken genome engineering applications, we present a concise description of the method and current application of the CRISPR/Cas9 system in chickens. Different strategies for delivering sgRNAs and the Cas9 protein, we also present extensively. Furthermore, we describe a new gesicle technology as a way to deliver Cas9/sgRNA complexes into target cells, and we discuss the advantages and describe basal applications of the CRISPR/Cas9 system in a chicken model.

## Introduction to editing methods in chickens

Chicken is a valuable model for various research areas, especially for avian transgenesis and genome editing. Today, the generation of genetically modified chickens (Mcgrew et al. 2004; Macdonald et al. 2012; Tyack et al. 2013; Song et al. 2014) is much more attainable than ever before, and there is a need for precise genome targeting. Specificity of chicken model is based on short generation times and large numbers of progeny, which facilitate achievement desired results. Similarity of pattern protein glycosylation to human, low biochemical complexity of native egg proteins, and cost-effective biopharmaceuticals production are one of the benefits of using chicken model.

Genome modification and engineering are based on the insertion, deletion, or replacement of genes to alter genetic information. To study gene function and production phenotypes, we have the tools to precisely edit the chicken genome. For birds, the application of the CRISPR (clustered regularly interspaced short palindromic repeats)/Cas9 (CRISPR-associated protein 9 system) system to avian somatic cells (Brown et al. 2003; Kobayashi et al. 2015; Daly et al. 2016) and tissues (Véron et al. 2015) has been reported. Additionally, specific genetic modifications of the chicken genome can be introduced by targeting primordial germ cells (PGCs) and by using these edited cells to produce gene-edited chickens.

PGCs are highly specialized cells, which first arise from the epiblast and are initially localized in the central disc of the pellucida area of stage X embryos (Eyal-Giladi and Kochav 1976). PGCs are located in the ventral surface of the pellucida. During embryo development, cells are translocated to the dorsal side of the hypoblast and subsequently migrate to the germinal crescent region at stage 4 H.H. according to Hamburger and Hamilton (1992). Once blood vessels form, PGCs enter the blood vessels between stages 10 H.H. and 12 H.H. and begin to circulate in the bloodstream. PGCs can be easy isolated from embryonic blood and used for further applications. Between stages 20 and 24 H.H., PGCs migrate into the gonad primordium, where they begin to differentiate into male or female gametes (Kuwana et al. 1986; Nakamura et al. 1991). In this stage, PGCs can be commonly acquired through gonad isolation and trypsinization. PGCs can transmit genetic information to next generations and are thus ideal for creating transgenic or gene-edited chickens (Bednarczyk et al. 2002; Bednarczyk et al. 2003; Van de Lavoir et al. 2006; Naito 2015; Chojnacka-Puchta et al. 2015; Sawicka et al. 2015; Oishi et al. 2016; Dimitrov et al. 2016). However, PGCs are characterized by their low ability to induce effective and persistent transfection. Additionally, there are several obstacles to this process, because germ cells are relatively transcriptionally quiescent and prone to switching off transgene expression (Seydoux and Braun 2006).

Development of efficient methods for culturing chicken PGCs without the loss of germline competency (Song et al. 2014; Naito 2015; Whyte et al. 2015; Lee et al. 2016) was a milestone that brought more possibilities for genetic modification and precise gene editing. The first transgenic birds were generated using PGCs isolated from chicken embryos (stage 11 H.H.) (Vick et al. 1993). Since then, both methods, modification of PGCs and rapid plasmid construction, have been improved in chicken transgenesis.

New transgenic technologies offer the opportunity to understand gene function and/or to modify the phenotype of animals according to defined production and scientific goals. Whole genome/transcript sequencing analysis and newly developed genome editing technologies have provided us opportunities to explore biological phenomena. In particular, the development of new molecular sequence-specific nucleases (SSNs), transcription activator-like effector nucleases (TALENs) or zinc-finger nucleases (ZFNs), and the powerful CRISPR/Cas9 technology (Bibkova et al. 2002; Christian et al. 2010; Jinek et al. 2012; Cho et al. 2013; Cong et al. 2013) has revolutionized the field of genome editing and enabled researchers to generate mutations and cut DNA in a very precise manner by activating double-stranded breaks (DSBs). TALENs and ZFNs used to recognize target sequence need specially designed proteins, which makes their synthesis expensive and difficult.

These nucleases can create DSBs in DNA to initialize repair pathways in cells, and these pathways can be used to knockout genes and support gene targeting. Eukaryotic cells mostly repair the break in two ways: through a NHEJ (nonhomologous end joining) repair pathway or less frequently, by a HDR (homology direct repair) pathway. NHEJ-repaired DSBs are usually imperfect, error-prone, and can react during the entire cell cycle. This can induce small insertions or deletions (InDel) in target genes and can be exploited to generate null mutation alleles. At the target site within an exon, InDel mutations generate frame shift mutations in both or one allele. HDR, the cellular direct repair mechanism of homologous recombination, generates perfect repair or precise genetic modifications when a repair DNA template is provided. The error-free results can be achieved in late S to G2 phases of the cell cycle (Zhao et al. 2017) during introduction of designed sequence changes.

The classical gene targeting by homologous recombination (HR) was reported by Schusser et al. (2013). They described the joining (J) gene segment of the chicken Ig heavy chain gene by HR in PGCs to obtain immunoglobulin knockout in chickens, but the efficiency of germline transmission was very low. The gene replacement or knock-in is rather less efficient than gene knockout; therefore, the HDR pathway is less powerful.

Advances in chicken germ cell biology and genome editing and our ability to generate germline chimeras might lead to some introduced mutations and will influence the biology and development of vertebrates (chicken model). This may allow investigation into the long-term consequences of transgenerational inheritance of genetic information.

## CRISPR/Cas9-mediated genome editing

The mechanism of the CRISPR/Cas gene editing technology developed from natural defense systems in bacteria against phage and plasmids. It has been adopted for genome editing in eukaryotic cells (Cong et al. 2013). This powerful CRISPR mechanism has been identified in six (I–VI) individual types and was further grouped into two classes (Wiedenheft et al. 2011; Wright et al. 2016; Jiang and Doudna 2017). The type II CRISPR system was validated experimentally in 2008 (Brouns et al. 2008) and is the most studied. This II CRISPR system uses only the Cas9 protein, which assists in the processing of crRNAs, binds, and cleaves the target DNA (Makarova et al. 2011). The CRISPR locus included the trans-activated CRISPR RNA gene (tracrRNA), Cas9 protein, spacer, and repeat CRISPR sequences. In this unique mechanism, the tracrRNA hybridizes to the crRNA, which contains a 20 nucleotide (nt) protospacer adjacent motif (PAM) element and complementary sequence to tracrRNA. They form the special dual RNA guide which directs Cas9 to recognize and cut a target sequence of DNA. The DNA endonuclease-Cas9 consists of two nuclease domains, HNH and RuvC. The HNH nuclease domain cleaves complementary DNA strands, while RuvC nuclease domain cleaves opposite DNA strands.

CRISPR/Cas9 technology is widely used in poultry and mammals (Li et al. 2013; Wang et al. 2014; Niu et al. 2014; Crispo et al. 2015; Savell and Jeremy 2017; Xu et al. 2018). In particular, there are less data on the in vivo effects of the CRISPR/Cas9 treatment in avian species, which requires further systematic studies.

The first study using a CRISPR/Cas9 system in chickens was published in 2015 and involved the electroporation of chicken embryos with plasmids encoding Cas9 and guide RNAs against the transcription factor PAX7. The results clearly demonstrated that this system was able to efficiently mediate gene editing in chicken embryos, indicating that it could be a valuable tool to study the molecular mechanisms regulating development in chickens (Véron et al. 2015). In subsequent studies, the CRISPR/Cas9 system has been used to knockout genes in chickens (Oishi et al. 2016; Zuo et al. 2016; Zhang et al. 2017). Oishi et al. (2016) generated ovomucoid homozygous offspring (G2) by crossing G1 mutant birds using cultured primordial germ cells (PGCs) and the CRISPR/Cas9 system. Using this strategy, they found deletions ranging from 1 to 12 base pairs (bp) in the OVM target site. In this study, three G0 germline chimeras were obtained, only two of which had a relatively high contribution from mutated donor sperm. Recently, Antonova et al. (2018) demonstrated that the CRISPR/Cas9 system with the cassette for HDR could be used in a chicken cell line to insert an *eGFP* gene into a genomic *GAPDH* locus. After adding G418 to the cells for selection, the efficiency of CRISPR/Cas9-mediated HDR was increased to 90%. They have inserted the eGFP gene after the GAPDH coding sequence under control of the chicken *GAPDH* promoter. However, in this study, the efficiency of gene knock-in was very low (targeting rate for gRNA2 was around 1.8% in DF1 cells).

Previous studies carried out on mammals, and chickens have indicated that the establishment of germline competent cells was the critical step for genome editing. Regardless of the target organism, all researchers using sequence-specific nucleases face similar challenges, confirmation of the desired on-target mutation, detection of off-target events (Zhang et al. 2018; Zischewskia et al. 2017), and, above all, investigation of the effects of the CRISPR/Cas9 treatment in vivo. Further development of editing technology using CRISPR/Cas9 should focus on solving these problems. A new available vesicle technology is an effective tool for genome manipulation and allows to overcome obstacles like obtaining efficient delivery of Cas9 and gene-specific single guide RNA (sgRNA) to all cell types and achieving fewer off-target effects. For a particular genome editing experiment, an optimal method for the delivery of the components of the CRISPR/Cas9 system is necessary.

## Strategies of delivery of CRISPR /Cas9 components

Different strategies are used to edit the genome by a CRISPR/Cas9 system (Liu et al. 2017). The easiest is the use of the same vector to express Cas9 protein and sgRNA (Morin et al. 2017). Using this approach, we can avoid the use of many transfection reagents that may disrupt and affect the efficiency of the whole CRISPR/Cas9 process. The next strategy is to introduce a mixture of the Cas9 mRNA and the sgRNA, while Cas9 mRNA will be translated to Cas9 protein in cells from the Cas9/sgRNA complex. The third strategy is to directly deliver into cells a mixture of the Cas9 protein and the sgRNA. All these approaches are used to edit chicken genes, however, with varying effectiveness.

A popular approach is based on application of a plasmid encoding Cas9 protein and sgRNA. The advantages of this strategy are simplicity, avoidance of multiple transfections, and enhanced stability. However, this approach also has limitations, such as more off-target effects and the necessity of delivering plasmid into the nucleus, which requires choosing the right method. The introduction of plasmids carrying Cas9 protein and the sgRNA sequence was performed via lipofection, polyethyleneimine (PEI), or electroporation methods. Zuo et al. (2016) designed three gRNAs to knockout the *C2EIP* gene and examined the efficiency of gene disruption in DF-1 chicken fibroblasts and chicken embryonic stem cells (ESCs). To evaluate the effects of this knockout in cells, they used a luciferase single-strand annealing (SSA) recombination assay, TA clone sequencing, and T7 endonuclease I (T7EI). The results of this analysis indicated that knockout efficiency was 27%. The same gene was subjected to disruption in chicken embryos. The recipients were injected with a PEI encapsulated CRISPR/Cas9 vector. A disruption of the *C2EIP* gene was generated in three of the 20 embryos (15% efficiency), as confirmed by the T7EI assay and TA clone sequencing.

Similar studies were conducted by Zhang et al. (2017). Three sgRNAs used to knockout the *STRA8* gene in DF-1 cells, and chicken ESCs were created. The Cas9/sgRNA plasmid was introduced into cells using the lipofection method. The efficiency of knockout in DF-1 cells and ESCs was 25% and 23%, respectively. In this study, PEI was also used to introduce the Cas9/gRNA plasmid into chicken embryos. Analysis by using a T7EI assay showed that the efficiency of *STRA8* gene knockout in the embryos was 12%.

Abu-Bonsrah et al. (2016) applied lipofection or electroporation to deliver sgRNA and the Cas9 protein into cells and chicken recipients. They generated *HIRA*, *TYRP*1, *DICER*, *MBD*3, *EZH*2, and six other knockouts in two cell lines (DF-1 and DT-40 cell lines) with similar effectivity (26–68%). The Cas9/sgRNA plasmid was also introduced into chicken embryos using the electroporation method. They edited the *DGCR8* gene in embryonic neural cells. In addition to the desired mutations, this direct procedure caused deformations in the heart and outflow tract in over 41% of individuals.

Another method for delivering sgRNA/Cas9 plasmids into chicken embryos is based on the transfection of PGCs with sgRNA and Cas9 plasmids, culturing the modified PGCs and injecting them into host embryos at the appropriate stage of development. Electroporation or lipofection has been used to transfect PGCs. Dimitrov et al. (2016) used the HDR strategy and CRISPR to target the heavy chain of the chicken immunoglobulin locus in PGCs. They modified PGCs with the Cas9/sgRNA plasmid using electroporation. After transfection, cells were selected with antibiotics to obtain stable transfectants. Confirmed clones were injected into recipient embryos to generate chimeric birds, which were raised to sexual maturity. After mating the chimeras, 13 chimeric roosters were obtained with germline transmission at an average level of 14.5%. This study demonstrated the first successful application of CRISPR/Cas9-assisted HDR of donor DNA in chickens. The same method of delivery Cas9/sgRNA was also applied in the Oishi et al. (2016) study. Cultured PGCs were transfected with the Cas9/sgRNA plasmid using lipofection. The sgRNA was designed to knockout the ovomucoid gene. PGCs containing the desired mutations were enriched using antibiotic selection. Genomic fragments including the sgRNA target sites were PCR amplified and sequenced. This analysis showed that the frequency of the desired mutation varied from 13% to 92%. These modified PGCs were injected into irradiated chicken embryos, which gave rise to healthy progeny. TA cloning analyses of the progeny indicated that two roosters exhibited *OVM* mutations with germline transmission at a level of 58%.

CRISPR/Cas9 components can be also directly delivered as mRNA for Cas9 and sgRNA into target cells. Genome editing in cells is initiated after expressing the Cas9 protein and forming the Cas9/sgRNA complex inside cells. The lower cytotoxicity and transient expression of Cas9 was demonstrated by Li et al. (2014) in cell lines and primary cells. Also, lower off-target effects and easy introduction into the cytoplasm to exert their effects are the main advantages of using this strategy. Unfortunately, mRNA exhibits low stability, which is a disadvantage of this approach. This strategy was used in combination with lipofection. Cooper et al. (2017) demonstrated an alternative to the methods for germ cells presented above, and used sperm to deliver gene editing vectors via sperm transfection-assisted gene editing (STAGE). Transfected sperm used for artificial insemination was obtained after introduction of Cas9 mRNA and sgRNA into cells using lipofection. The targets used to disrupt were a previously integrated *GFP* gene and the endogenous doublesex and mab-3-related transcription factor 1 (*DMRT*1) gene. The highest efficiency of this method was 26%. STAGE is an effective method to generate heterozygous and homozygous gene-edited birds in a single generation.

Another technique used to edit genes is to deliver sgRNA and Cas9 protein as a Cas9/sgRNA ribonucleoprotein (RNP) complex. The characteristic features are high efficiency of gene editing, reduction of off-target effects and lower toxicity. Furthermore, promoter selection and codon optimization are not required. In the Lin et al. (2014) study, the Cas9/sgRNA RNPs in combination with nucleofection were used to edit the *EMX1* gene in HEK 293 T cells, human primary neonatal fibroblasts, and human embryonic stem cells. The efficiency of HDR-mediated genome editing was at levels up to 38%.

### Novel gesicle technology

Recently developed technology is based on a combination of Cas9/sgRNA RNP with derived nanovesicles, called gesicles. They are produced by a special modified cell line 293 T (Gesicle Producer Cell Line, Takara Bio, Shiga, Japan) via co-overexpression of the packaging-mix components, which include a nanovesicle-inducing glycoprotein and a protein for cell-surface display that mediates binding and fusion with the cellular membrane of target cells. Produced gesicles contain target-specific Cas9/sgRNA RNP complexes and fuse to target cells. Next, the Cas9/sgRNA complex is released and translocated to the nucleus to perform site-specific gene editing. Compared to previous methods, this system has two important advantages. First, the CRISPR/Cas9 complex is delivered into target cells via gesicles, which reduce the toxicity to cells. Early studies have warned of the frequent off-site effects in the CRISPR/Cas9 system (Fu et al. 2013; Hsu et al. 2013; Mali et al. 2013a, b). The second benefit of nanovesicle systems is that they lead to fewer off-target effects, due to the short life span of the Cas9 endonuclease in mutated cells. Hsu et al. (2013) suggested that consistent expression of Cas9 can cause unwanted cleavage events at off-target sites. However, reducing the amount of transfected DNA can lead to a depletion of on-target cleavage. The study showed that the amount of complexed Cas9 and sgRNA should be titrated to optimize the on-site and off-target ratio of cleavage. A comparison of nanovesicle technology and plasmids as a two method system to deliver Cas9/sgRNA to edit the *EMX1* gene in HEK 293 T cells has been demonstrated by scientists from Takara (Web document Tech Noten.d). They treated cells with gesicles including Cas9/sgRNA complexes or transfected with plasmids containing the Cas9 gene sequence and gene-specific sgRNA. Four potential off-target loci were chosen. The presence of indels was detected with the use of a resolvase digestion system. As assumed, delivery of gesicles led to no observable indel formation at the off-site locus, but plasmid transfection showed significant indel formation at this locus. This developed universal tool for genome modification provides a direct, rapid, and transient method for delivering active genome-modifying proteins to target cells.

To date, this system has been used only with human induced pluripotent stem (hiPS) cells and different mammalian cell lines (HEK293, HeLa, MCF7, NIH3T3, HT1080, CHOK1, RPE, Raji, Jurkat, HepG2, and KBM7). We performed the first preliminary study to apply the gesicle system to deliver a Cas9/sgRNA complex into hard-to-transfect and very sensitive chicken primordial germ cells (unpublished data). Our results confirmed that this system was suitable for PGCs and may be a powerful technique for genome manipulation in chicken PGCs. The new method can reduce off-target events and eliminate problems with constitutive expression of the Cas9 protein in target cells. Delivery of the Cas9/sgRNA complexes to target cells using gesicles is efficient and leads to reduced off-target effects compared to other methods of Cas9 and sgRNA delivery.

## Advantages and basal applications of the CRISPR/Cas9 system

While different strategies allow researchers to choose the appropriate procedure, the CRISPR/Cas9 system has many advantages and unfortunately some weaknesses. Editing methods are difficult and inefficient for chickens, and CRISPR seems to be a simpler, cheap, and more effective tool, which is why an appropriate system for carrying out targeted mutagenesis to obtain transgenic chickens is being sought. Combining the appropriate strategy of delivering Cas9 and sgRNA with the appropriate cells and database tools for CRISPR can result in high efficiency and success of the editing process.

In comparison to ZFNs and TALENs, target designs in CRISPR/Cas9 are easy and inexpensive. The gRNAs can be designed simply against nearly any sequence of the genomic target in the genome because target specificity relies on ribonucleotide complex formation and not protein/DNA recognition. Computational tools help in the design of sgRNAs. We can thus predict guide specificity and try to reduce off-sites effects. Also, successful sgRNAs should have strong on-target activity (Wilson et al. 2018). The Cas9/sgRNA complex can be used for specific genomic loci, leading to DNA DSBs. Most current off-target prediction tools are based on rules derived from earlier experiments. These rules can be complicated and may require scanning the sequence of a genome for mutations at sites with nucleotide similarity to the gRNA target sequence. Xiao et al. (2014) designed the CasOT-searching tool, which could indicate potential off-targets and effectively identify those sites throughout the genome. Also, the right choice of delivery system and proper type of CRISPR/Cas9 can reduce off-sites effects. For instance, nonviral delivery systems indicate a smaller amount of off-target effects than viral delivery systems.

CRISPR technology can be easy to use and efficient if applied to analyze the influence of the interactions between genetic differences and gene expressions. Modifications can be directly introduced by injecting RNAs encoding the Cas protein and gRNA into developing embryos or cells. Cho et al. (2014) indicated that raising the gRNA concentration could improve gene knockout efficiency close to 33% in a co-transfection system.

Furthermore, multiplex gene mutations are possible with Cas9 nuclease, and it is a very important feature of the CRISPR/Cas system. Multiple genes can be simultaneously mutated by expressing multiple gRNAs. Wang et al. (2013) reported using the CRISPR/Cas9 technology to successfully and simultaneously introduce mutations in five genes in mouse ES cells. In addition, Xie and Yang (2019) demonstrated an efficient polycistronic tRNA-gRNA (PTG) method to produce many tRNAs from a single gene transcript. The PTG method is based on endogenous tRNA processing and has been used successfully for multiplex genome editing in various organism from microbial species to vertebrates. Furthermore, CRISPR can be used to knockout genes and replace them using other gene therapy systems (Zhang et al. 2015). Deleting or inverting the target region facilitates the use of the two sgRNAs that flank the desired genomic region. Due to the targeted action and the precisely refined nuclease cleavage site, CRISPR/Cas9 is the perfect tool for effective knockout and knock-in.

The knockout system has revolutionized the research field of functional genomics by allowing the analysis of specific gene functions (Schusser et al. 2013; Dimitrov et al. 2016; Oishi et al. 2016). Cheng et al. (2019) investigated the effect of the chicken TANK-binding kinase 1 (*TBK1*) gene deletion on chSTING-mediated (chicken stimulator of interferon gene) interferon-beta (IFNβ) production in chicken cells. The ch*STING* was identified as an effective IFN mediator that interacted with ch*TBK1*, and these results were very important in the study of gene function. They created a chTBK1-knockout DF-1 cell line called DF-1-TBK1-C3 using a CRISPR/Cas9 system to efficiently target chicken cells and verified that ch*TBK1* was necessary for ch*STING*- mediated IFN regulation.

Knockout techniques have been developed and utilized for disruption of the targeted avian gene or locus in the agricultural industry. Generation of superior genetic lines is one of the major poultry research directions. Gene-edited chickens were produced after using TALENs (Park et al. 2014) or CRISPR/Cas9 technology (Oishi et al. 2016) to demonstrate that disruptions of ovalbumin and ovomucoid genes had the potential to produce low allergenicity in eggs, which allowed a reduced immune response in egg white sensitive individuals. This is very important for both food products and the vaccine industry. Lee et al. (2019) presented the results of using CRISPR/Cas9 adenoviral vector directly injected into the quail blastoderm. The offspring obtained from quail chimeras had mutations in melanophilin (MLPH) gene. Development of genome-edited poultry is one of the expected achievements and challenges of agriculture.

To improve economic traits of animals, including in sheep, cattle, rabbits, goats, and pigs, the knockout systems have been also used (Proudfoot et al. 2015; Guo et al. 2016; Bi et al. 2016). In these studies, the myostatin (MSTN) gene was disrupted using targeted editing technology, which led to enhanced formation of skeletal muscles, and this could be beneficial for meat production. The first approach to knockout the MSTN gene in chicken DF-1 cells has been demonstrated by Lee et al. (2017). The authors used the Cas9-D10A nickase of mutated CRISPR/Cas9 to efficiently modulate the MSTN gene.

Gene editing can be also a useful tool to generate disease-resistant chickens. The first attempt to use CRISPR/Cas9 mediated knockout of the ALV (avian leukosis virus) receptor locus was reported in DF-1 cells by Koslová et al. (2018). They introduced frame-shifting indel mutations into TVA, TVC, and TVJ loci encoding receptors for the A, C, and J ALV subgroups. This study was a crucial step in the development of virus resistant chickens. This is also important from the point of view of the poultry farm workers, which are exposed to different biological hazards include inter alia ALV (Feng and Zhang 2016) or H5N1 and H7N9 viruses (Yang et al. 2016). Generation of disease-resistant chickens will be a milestone in poultry production and definitely impact on increasing of human work safety.

The use of chickens as bioreactors provides an alternative therapeutic approach, which greatly benefits human health (Zhu et al. 2005). Previous studies demonstrated using modified chickens to produce, for example, antimicrobial peptides (Liu et al. 2015), monoclonal antibodies for breast cancer therapy (Oishi et al. 2011), lysozyme (Cao et al. 2015), epidermal growth factor (Park et al. 2015), and human cytokine interferon α2a (Herron et al. 2018). Recently, Oishi et al. (2018) has proven that the CRISPR/Cas9 system can be successfully used to produce recombinant proteins in egg whites. They knocked in human IFN-β into the chicken ovalbumin locus in PGCs and generated KI hens that produce this therapeutic protein. This finding will open many avenues for implementing biopharmaceutical production in chicken eggs.

CRISPR/Cas9 gene editing is another opportunity to better understanding biology of herpesviruses and associated with them, virus-induced oncogenesis. Zhang et al. (2019) first reported successful and effective using of CRISPR technology to make targeted mutations in situ into the viral genome of Marek’s disease virus (MDV) – transformed lymphoblastoid cell line (LCL) for studies of viral latency and interactions between a virus and its host. The same gRNAs were used for precise editing of the viral gene phosphoprotein 38 (*pp38*) in infected primary chick embryo fibroblast (CEF) and to insert the GFP gene into the viral *pp38* locus in MDV transformed cell line (HP8 cells) by NHEJ pathway. Development of gene editing technology is very rapid, and more applications should be evaluated in the near future.

## Conclusions

Over the years, significant progress has been made in achieving the targeted effects in chickens using genetic engineering tools. Recently developed CRISPR/Cas9 system is invaluable tool in variety of the disciplines and has facilitated generating gene-edited chickens with high effectiveness.

There are different strategies to edit genes via CRISPR/Cas9 technology and various methods to deliver CRISPR/Cas9 components into target cells. The demonstrated novel gesicle strategy allows efficacious enrichment of manipulated chicken PGCs and lays the foundation for future generations of knockout chicken lines in spite of its application being only in the preliminary stages. Application of a CRISPR/Cas9 system can lead to better understanding of the development of chickens, and it is a robust tool for chicken genome editing. The use of modified CRISPR/Cas9 PGCs fundamentally broadens the impact of bioengineering methods to improve chicken productivity and health. Further development of this genome editing technology is expected and provides the opportunity for production of improved knock-in chickens for agricultural industries, biotechnology, and biomedical applications.

In conclusion, CRISPR techniques have some advantages in relation to animal improvement: technical and competitive benefits. Genome-edited chickens can be produced using a CRISPR-controllable system, which is similar to what could be obtained through natural mutations and conventional breeding, although it is directed, more precise, and quicker. There are many concepts proposed in chickens for food and must be a suitable level of regulation to maintain consumer confidence (Tizard et al. 2019). It is worth to notice that the Court of Justice of the European Union on 25 July 2018 decided that organisms generated by the direct mutagenesis techniques are genetically modified organisms (GMOs), within the meaning of the Directive 2001/18/EC (Statement by the Group of Chief Scientific Advisors 2018). However, the animal biotech sector in Europe could have a new innovation tool and that will a competitive advantage.
